# Heart monitoring using left ventricle impedance and ventricular electrocardiography in left ventricular assist device patients

**DOI:** 10.1186/s12938-015-0019-3

**Published:** 2015-03-21

**Authors:** Keun Her, Chi Bum Ahn, Sung Min Park, Seong Wook Choi

**Affiliations:** Department of Cardiovascular and Thoracic Surgery, Soonchunhyang University Hospital, Bucheon-si, South Korea; Department of Mechanical and Biomedical Engineering, College of Engineering, Kangwon National University, 192-1 Hyoja-Dong, Chuncheon-si, South Korea; School of Medicine, Kangwon National University, Chuncheon-si, South Korea

## Abstract

**Background:**

Patients who develop critical arrhythmia during left ventricular assist device (LVAD) perfusion have a low survival rate. For diagnosis of unexpected heart abnormalities, new heart-monitoring methods are required for patients supported by LVAD perfusion. Ventricular electrocardiography using electrodes implanted in the ventricle to detect heart contractions is unsuitable if the heart is abnormal. Left ventricular impedance (LVI) is useful for monitoring heart movement but does not show abnormal action potential in the heart muscle.

**Objectives:**

To detect detailed abnormal heart conditions, we obtained ventricular electrocardiograms (v-ECGs) and LVI simultaneously in porcine models connected to LVADs.

**Methods:**

In the porcine models, electrodes were set on the heart apex and ascending aorta for real-time measurements of v-ECGs and LVI. As the carrier current frequency of the LVI was adjusted to 30 kHz, it was easily derived from the original v-ECG signal by using a high-pass filter (cutoff: 10 kHz). In addition, v-ECGs with a frequency band of 0.1 – 120 Hz were easily derived using a low-pass filter. Simultaneous v-ECG and LVI data were compared to detect heart volume changes during the Q-T period when the heart contracted. A new real-time algorithm for comparison of v-ECGs and LVI determined whether the porcine heartbeats were normal or abnormal. Several abnormal heartbeats were detected using the LVADs operating in asynchronous mode, most of which were premature ventricle contractions (PVCs). To evaluate the accuracy of the new method, the results obtained were compared to normal ECG data and cardiac output measured simultaneously using commercial devices.

**Results:**

The new method provided more accurate detection of abnormal heart movements. This method can be used for various heart diseases, even those in which the cardiac output is heavily affected by LVAD operation.

## Introduction

Ventricular assist devices (VADs) were developed to improve survival in patients with advanced heart failure [1-5]. The use of VADs improves survival rates, and is in this respect second only to heart transplantation [4,5]. VADs are commonly used as a bridge-to-transplant measure, but their use as destination therapy is increasing due to improved reliability and patient survival [5]. As the number of left ventricular assist device (LVAD) patients has increased, heart conditions such as arrhythmia have been detected in patients using VADs [6-9]. Ziv et al. suggested that the arrhythmia in these patients may be caused by the VADs [7]. Bedi et al. also reported lower survival rates in VAD patients who experienced arrhythmia within several weeks after LVAD implantation [8]. Refaat et al. showed that an implantable cardioverter-defibrillator (ICD) can increase the survivability of LVAD patients by properly treating critical arrhythmia [9]. However, the majority of commercial VADs do not include a heart-diagnosis function [10].

While electrocardiograms (ECGs) are necessary for diagnosis of arrhythmia, the additional leads and electrodes from the ECG device are uncomfortable for a patient who is already connected to a VAD. In addition, the VAD may interfere with the ECG, preventing accurate monitoring of the heart [11-13]. Some VADs circumvent the noise and other complications by connecting electrodes to the heart or subcutaneous tissues [14,15]. However, such electrodes are closer to the heart and the direction of measurement is altered, resulting in ECG recordings that deviate from standard measures, in both waveform and magnitude [14]. Ventricular electrocardiograms (v-ECGs) taken directly from the heart rarely show noticeable differences between normal and abnormal heart activities, even with the occurrence of severe abnormalities, such as premature ventricle contraction (PVC) [15]. Such limitations make it difficult to use v-ECGs for diagnosis of arrhythmia. The use of left ventricle impedance (LVI) has previously been suggested as a means of monitoring the heart in VAD patients [16]. LVI is measured by connecting electrodes to the cardiac apex and the aorta, and allows monitoring of changes in left ventricular volume. However, it cannot determine whether a change in ventricle volume is caused by natural heart muscle contraction and relaxation or by an inflow change resulting from the LVAD. In the case of a pulsatile LVAD, using LVI signals alone to monitor the heart is difficult due to the severely irregular blood inflow changes.

It is possible to record v-ECGs simultaneously with LVI, which is expected to provide valuable data for diagnosing arrhythmia. The increment of LVI during the R-T period can be derived from the v-ECGs. In addition, the changes in LVI signals during systole can be predicted under the assumption that the heart contraction and LVAD inlet pressure are stable with no changes caused by abrupt conditions or physiological factors. Therefore, discrepancies between the predicted impedance and the measured LVI can be interpreted as changes in heart performance since the initial recording. Using this comparison, we have successfully detected reduced heart performance coinciding with a PVC on an ECG.

In this study, LVI and v-ECG data were collected using electrodes linked to the hearts of three pigs connected to VADs. The predicted increases in LVI were calculated under simple conditions when the decrease in ventricle blood volume occurred due to heart co1ntraction during the R-T period and LVAD inflow. In addition, the R-T period and LVAD inflow period were determined from v-ECGs and the LVAD controller. During the experiment, multiple PVCs were observed using conventional ECGs. When the PVCs occurred, the measured LVI changes during R-T periods were lower than the predicted values. The results of this experiment confirm that the LVI decrements during the R-T period coincided with abnormal heartbeats, which could not be observed using v-ECGs or LVI alone.

## Materials and methods

### v-ECG and LVI measurement method

**Figure 1:**
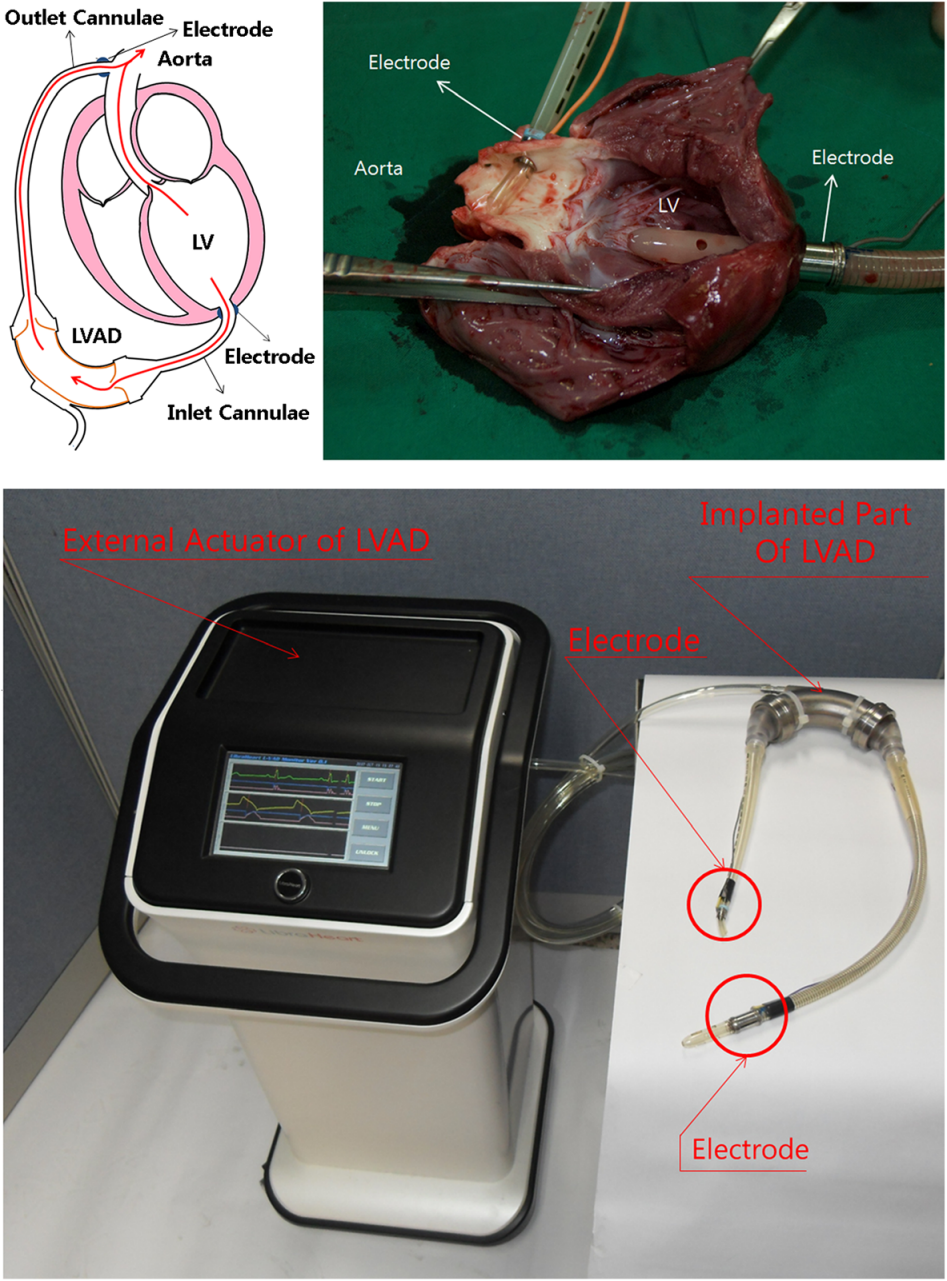
**Placement of electrodes for measurement of v-ECGs and LVI in the LV (upper) and pulsatile left ventricular assist device (lower).**

The electrodes were placed at the inlet and outlet of the VAD, in contact with the apex and aortic tissue, as shown in Figure 1. The electrodes were made of stainless steel with smooth surfaces to prevent thrombogenesis and tissue adhesion. Although separate animal experiments showed neither thrombogenesis nor tissue adhesion for over 45 days, long-term clinical trials may be needed to improve the performance and safety of the electrodes [16]. In this study, the electrode material had no impact on v-ECG or LVI measurements, and no measurement-induced damage or deformation of the electrodes occurred.

For v-ECGs, a reference electrode was placed on the aorta, while the measurement electrode was placed on the apex. This method has a similar direction of measurement to aVF in ECG measurements, but with different polarity and magnitude because the implanted electrodes are closer to the heart than the external electrodes used in conventional ECGs. The range of measurement of the v-ECGs was 0.1 – 120 Hz, and the gain was 1000, which are similar values to those of conventional ECGs. The amplified signal was saved in 16-bit resolution through an analog-to-digital converter (ADC) with a sampling rate of 250 Hz. LVI was measured by running a high-frequency current at 30 kHz at 0.1 mA through the electrodes placed on the apex and the aorta. LVI values were highest at the end of ventricular contraction or influx into the VAD, and lowest before heart contraction.

**Figure 2:**
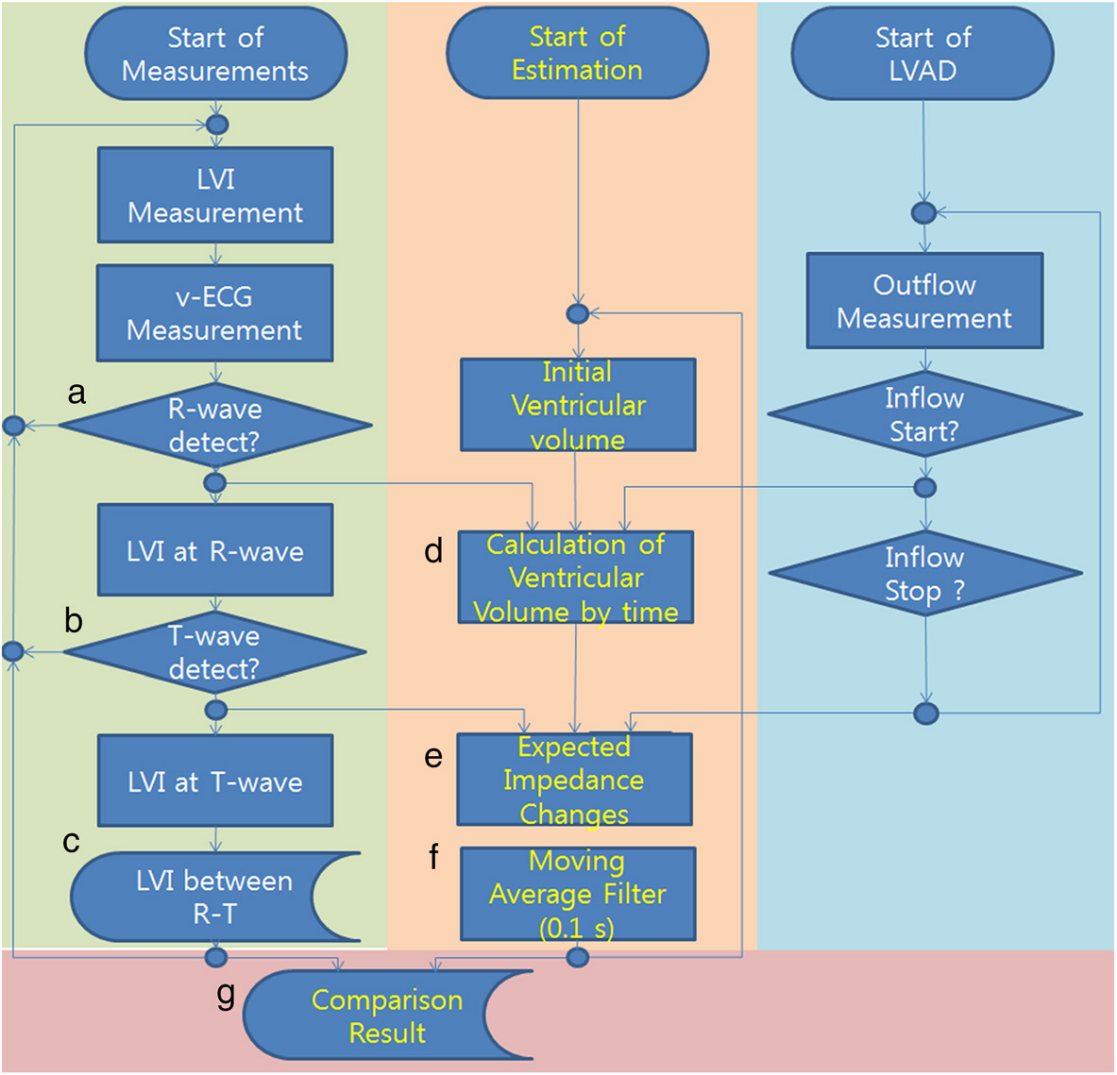
**Algorithm for (left) detection of R-T impedance increases, (right) VAD operation conditions, and (middle and bottom) comparison of R-T impedance and prediction. (a)** the R-wave detection in v-ECG, **(b)** the T-wave detection, **(c)** the calculation of LVI change during R-T period, **(d)** the prediction of volume change from v-ECG and LVAD inflow, **(e)** the impedance prediction, **(f)** the result of low-pass filter and **(g)** the comparison of the predicted value and the measured.

Figure 2 shows the algorithms for real-time analysis of v-ECGs and LVI measurements from a LVAD to monitor changes in heart activity. The left side of the algorithm shows the following sequence: (1) measure the v-ECG and LVI; (2) use the same algorithm to measure R-wave and T-wave; then (3) derive the change in LVI in the R-T period. The middle section shows the algorithm for calculating the change in impedance using the onset times of R- and T-waves, cardiac output (CO), and the duration and volume of influx into the LVAD. The right section shows the algorithm for sending the LVAD blood influx time and volume data to the impedance calculating algorithm [17]. Finally, abnormal pulses are distinguished by comparing the predicted impedance to the LVI value in the R-T period.

Measured v-ECGs show a negative R-wave value due to the inward sodium current of heart cells at depolarization prior to contraction. The T-wave has a positive value on the v-ECGs before relaxation due to the calcium and potassium current at repolarization. As shown in Figure 2 (a), the Tompkin method, which is used to find the QRS-complex in ECGs, is used to identify the R-wave from v-ECGs [18]. In Figure 2 (b), the T-wave is defined as the peak that occurs 50 – 450 ms after the R-wave and with a positive value greater than the threshold. (Other hemodynamic parameters are measured and compared to assess the accuracy of R- and T-waves, such as CO values determined using an ultrasonic flow meter and arterial blood pressure (ABP) using a monitor. The difference in R-wave detection time between v-ECGs and CO values was 16  ±  25 ms, and the difference between v-ECG and ABP was 22  ±  31 ms.)

During the R-T period, the decrease in LV volume is determined by blood flow into the LVAD and the aorta through the aortic valve. The LV volume decrease can be measured by the change in LVI during the R-T period. The average inflow of a LVAD can be estimated using the LVAD’s operating conditions and the total blood outflow from the LV can be measured by the thermodilution method. Changes in cardiac impedance are also affected by heart outflow. The blood flow through the aortic valve can be obtained by subtracting the total blood outflow of the LVAD from the change in cardiac volume during the R-T period. In addition, the blood flow through the aortic valve can be measured using an ultrasonic blood flow meter. However, measurement using a blood flow meter is possible for only a few minutes because the ascending aorta is an unsuitable location for an ultrasonic flow-meter probe due to the narrow thoracic cavity and the complexity of the vessels. Outside the R-T period, when heart contraction does not occur, LV inflow through the mitral valve can also affect the volume and impedance of the LV and make analysis of LVI difficult because no appropriate method for measurement of LV inflow is available. Therefore, changes in LVI outside the R-T period were not considered in this study.

LVI values are obtained at R- and T-waves and then the differences between these values are used to calculate the LVI changes, as shown in Figure 2 (c). To estimate heart activity, it should be determined whether the differences between the two LVI values were due to LVAD inflow or outflow through the aortic valve, ejected by heart contraction. The contraction phase can be recognized by the R-T period on v-ECGs, and the correlation between the resulting decrease in ventricular volume and increased heart impedance is well known [16]. During the R-T period, the magnitude of the decrease in volume can be predicted from the measured LVI; this decrease in volume can be separated into the amount of blood exiting the ventricle through the aortic valve and that entering the LVAD, as shown in equation 1.

1 $$ \mathrm{L}\mathrm{V}\ \mathrm{volume}\ \mathrm{decreas}{\mathrm{e}}_{\mathrm{R}\hbox{-} \mathrm{T}\_\mathrm{Period}}={\displaystyle {\int}_{\mathrm{R}\hbox{-} \mathrm{wave}}^{\mathrm{T}\hbox{-} \mathrm{wave}}\left(\mathrm{F}\mathrm{l}\mathrm{o}{\mathrm{w}}_{\mathrm{Aortic}\ \mathrm{V}\mathrm{alve}}(t)+\mathrm{inflo}{\mathrm{w}}_{\mathrm{VAD}}(t)\right)}\kern0.5em dt $$

Here, the average VAD inflow can be calculated from VAD operating conditions [19]. The total average flow can be measured by subtracting the inflow to the VAD from the flow measured by thermodilution. The increase in equation 1 during the R-T period can be described by equation 2, which is composed of total average flow and average inflow values.

2 $$ \mathrm{L}\mathrm{V}\ \mathrm{volume}\ \mathrm{decreas}{\mathrm{e}}_{\mathrm{R}\hbox{-} \mathrm{T}\_\mathrm{Period}}=\frac{\left( TotalFlow\mathit{\hbox{-}} Inflo{w}_{VAD}\right)}{HR}+\mathrm{Inflo}{\mathrm{w}}_{\mathrm{VAD}}\left({t}_{R\mathit{\hbox{-}}T}\right) $$

The total average blood flow or thermodilution cannot be measured continuously; however, if it is assumed to be maintained at a stable value, it can be used to predict LV volume and LVI change by using equation 2. In addition, after signal processing of the predicted values with a 0.15-s moving average filter, as shown in Figure 2 (f), the filtered values can be compared with measured LVI to find changes in total average blood flow. In Figure 2 (g), when the LVI change measured during the R-T period fell below 50% of the predicted value calculated after the end of a T-wave, the difference was attributed to abnormal performance of the heart.

### *In vivo* analysis

Animal experiments using three pigs (Yorkshire) weighing 35 – 40 kg were performed with the approval of the Institutional Review Board of Kangwon National University and in compliance with the Ethics and Regulations for Animal Studies. The animals were placed on the operating table in the supine position, and 10 mg/kg intramuscular ketamine was injected to induce anesthesia. Anesthesia was maintained during the surgical procedure and the sensor-signal-monitoring period by a constant supply of 1 – 2% inhaled isoflurane through an endotracheal tube. The inlet cannula (28 Fr; Medtronic Inc., Minneapolis, MN) was connected to the LV apex, and the outlet cannula (16 Fr; Medtronic Inc.) was connected to the ascending aorta through a median sternotomy. Mean ABP was 47.7  ±  11.0 mm Hg before LVAD perfusion and 48.7  ±  8.3 mm Hg during LVAD perfusion, mean heart rate (HR) was 86.7  ±  14.4 beats/min before LVAD perfusion and 92.3  ±  4.7 beats/min during LVAD perfusion, mean CO was 3.4  ±  0.4 L/min before LVAD perfusion and 1.9  ±  0.6 L/min during LVAD perfusion, and mean LVAD outflow was maintained at 1.3  ±  0.3 L/min. In all experiments, the animals survived for 3 h after starting the LVAD operation and were then euthanized by an overdose of anesthetic.

The inlet and outlet cannulas of the LVAD (LibraHeart I; LibraHeart Inc., Chuncheon, Korea) were connected to the animal’s LV and aorta, respectively, and the other ends of the cannulas were connected to the inlet and outlet ports of the pump for use as a pulsatile LVAD [15,19]. The v-ECGs were measured using electrodes set on the surfaces of the inlet and outlet cannulas; the electrodes were in contact with the heart muscle and aorta wall when the cannulas were connected [15,16,19]. v-ECG measurement on the LV is similar to a unipolar-lead ECG measurement, except that the electrode contacts the cardiac muscle directly; the measurable range is limited, and the pattern of waveforms is very simple. LibraHeart I has an amplifier for the v-ECG with an amplification rate of 250×, analog output, and an analog-to-digital converter (ADC; 250/s, 16-bit). LibraHeart I used the measured v-ECG to detect the R-wave using Tompkin’s algorithm and to maintain the LVAD in counterpulsation mode with the heart. Through the analog output, the measured v-ECG was transmitted to an external data acquisition device (NI USB-6259; National Instruments, USA) that could save measured v-ECGs with other vital signs from a patient monitor (BPM1200; Bionics Inc., Korea).

Three additional electrodes were attached to both forelegs and the left rear leg of the animals to compare the v-ECG measurements with those of conventional ECGs (BPM1200; Bionics Inc., Korea), and ABP was measured at the carotid artery. After connecting the cannula, v-ECG data were measured by the amplifier of the LVAD. All measured data were immediately sent to a data-acquisition device (PXI6224; National Instruments Corp., Austin, TX) through the analog channel.

## Results

**Figure 3:**
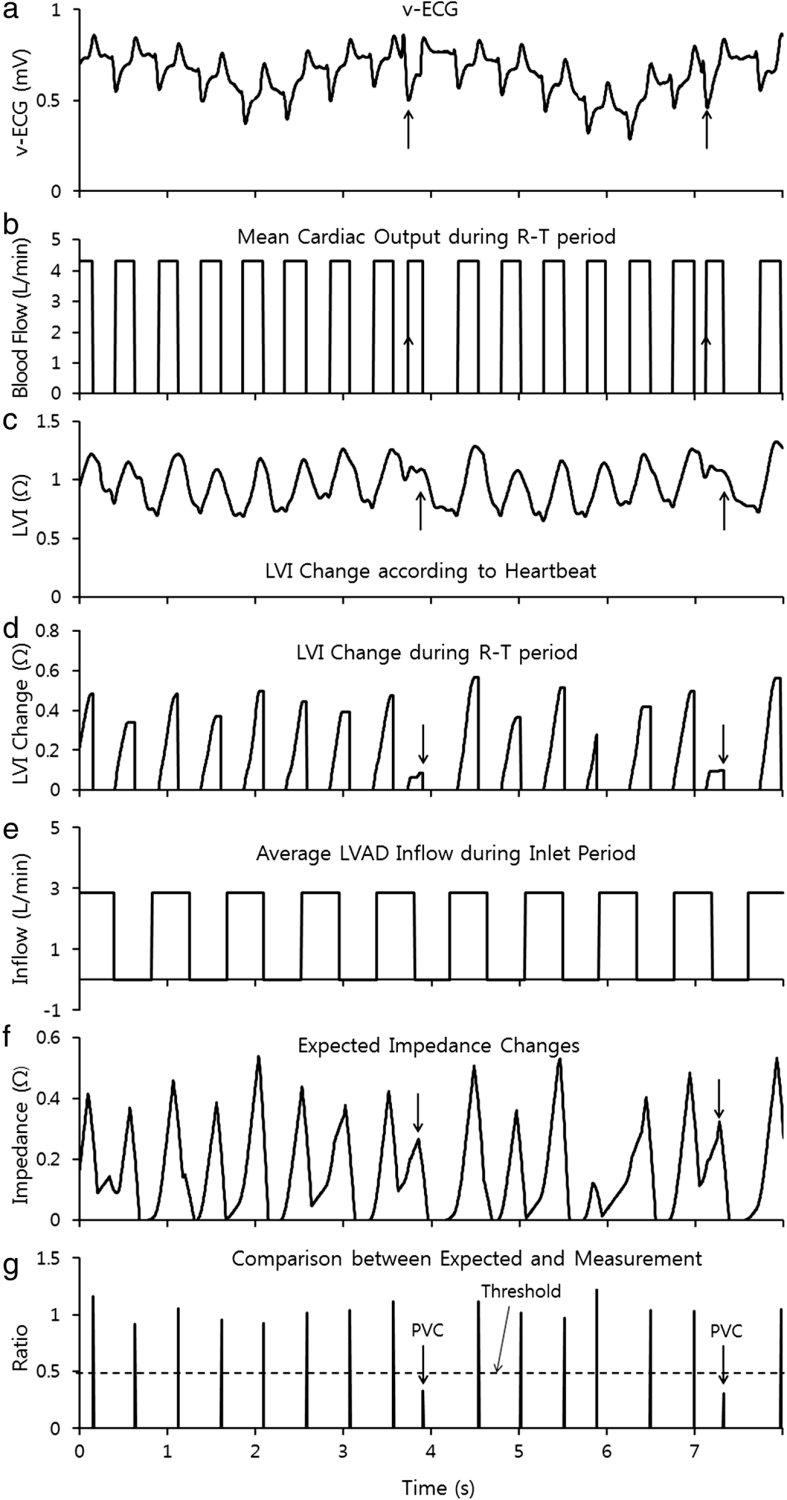
Signal processing for the detection of abnormal heart beats **(a)** the v-ECG recorded from the in-vivo experiment, **(b)** the R-T period determined by the v-ECG in Figures 2 a, b, **(c)** the measured changes in LVI, **(d)** the magnitude of change in LVI during the R-T period, as measured in Figure 2 c, **(e)** the period during which blood flowed into the VAD, **(f)** the predicted increase in impedance during mean blood flow and inflow from the heart when the R-T period and increase in volume appeared to predict high impedance and **(g)** comparison of the predicted impedance in Figure 3 f and the actual LVI increase in Figure 3 d.

**Figure 4:**
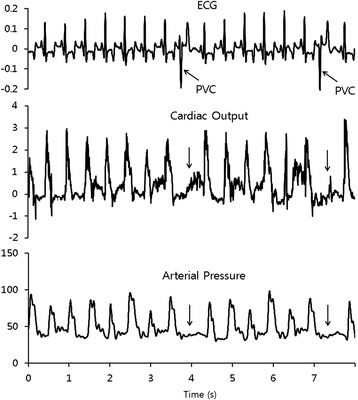
**Simultaneous measurement of ECG, CO, and ABP.**

The v-ECG in Figure 3 (a) was obtained from the in-vivo experiment. It showed distinct R-waves and T-waves on the v-ECG whether the heart beat were normal or abnormal. However, many abnormal heart beats could not be identified with only the v-ECG; although these abnormal signals could be found by analyzing the conventional ECG signal measured at the same time (Figure 4 (a)). The R-T period could be calculated with the v-ECG by measuring the delay from the R-wave to the T-wave according to the algorithm shown in Figure 2 (a) and (b). The R-T period is shown in Figure 3 (b) and the y-axis of Figure 3 (b) shows the average blood flow during the R-T period. Outside of the R-T period, the aortic valve was assumed to be closed and the blood flow ceased.

The LVI shown in Figure 3 (c), measured simultaneously with the v-ECG, did not show distinct aspects during heart contraction since the volume of the ventricle decreases not only by the heart muscle’s contraction but also by the suction of the LVAD. However, the LVI differences at the R-wave and the T-wave can show ventricular volume changes during heart contraction as shown in Figure 3 (d). The average inflow amount of the LVAD and inflow period were obtained from the LVAD controller as shown in Figure 3 (e).

The predicted heart volume in Figure 3 (f) can be calculated using the values of the average blood flow and LVAD inflow assuming those values remain in stable state. It can be compared with the measured LVI changes and predicted heart volume as shown in Figure 3 (g). When the measured LVI was less than half of the predicted value, the heart beat was classified as abnormal because the volume change at the heart beat was smaller than normal. Heart beats classified as abnormal were compared with a conventional ECG, cardiac output (CO) and arterial blood pressure (ABP) in Figure 4. The conventional ECG in Figure 4 (a) shows that most abnormal heart beats were PVC. CO and ABP show that the blood flow through the aortic valve at PVC was significantly smaller than for other normal beats. Table 1 verifies the reliability of the new comparison by showing distinct different values at normal heart beat and at PVC. VAD intake caused a relatively small increase in LVI compared to the heart’s contraction. This appears to be because the VAD intake of blood does not reduce the volume of the LV by inducing blood inflow from the left atrium (LA).

**Table 1:** **LVI increase during R-T period**

	**LVI increment**	**Comparison between lvi (r-t period) & expectation (%)**
	**(Ohm, r-t period)**	
**Normal**	#1:	
	0.277 ± 0.107 (n = 1041)	100.7 ± 11.2
	#2:	
	0.395 ± 0.159 (n = 1467)	111.6 ± 13.8
	#3:	
	0.424 ± 0.084 (n = 1693)	105.4 ± 11.5
	Average	
	0.365 ± 0.117 (n = 4201)	105.9 ± 12.2
**Abnormal (pvc)**	#1:	
	0.098 ± 0.051 (n = 110)	34.7 ± 17.8
	#2:	
	0.127 ± 0.067 (n = 51)	33.7 ± 10.9
	#3:	
	0.047 ± 0.028 (n = 36)	20.0 ± 8.5
	Average	
	0.091 ± 0.049 (n = 197)	29.5 ± 12.4

## Discussion

This study was performed to detect abnormal contraction of the heart by observing the impedance of the LV. During these experiments, abnormal contractions were observed during asynchronous perfusion with a pulsatile VAD. No previous study has indicated that the pulsatile LVAD can induce abnormal heartbeat in asynchronous mode. However, such conditions alter the load on the heart, and changes in cardiac load increase the risk of arrhythmia. In cases in which arrhythmia is observed, an implantable cardioverter-defibrillator (ICD) could be applied to the LVAD patient to treat advanced critical arrhythmia. In addition to the change in load, sudden stretching of the cardiac muscle may also stimulate the muscle and cause arrhythmias, such as PVC. Although these experiments did not indicate a close correlation between stretching and PVC occurrence ratio, it is possible that these PVCs may be due to irregular volume changes caused by a pulsatile LVAD.

Previously, LVIs were measured using continuous flow VADs. In previous studies using continuous flow VADs, the LVI and the amount of blood in the ventricles were considered to be correlated. Unlike these previous experiments using continuous flow VADs, those involving pulsatile VADs showed relatively complex changes in LVI patterns. As the measurement location and methods used in this study were identical to those in previous studies, the same correlation was expected to be present using pulsatile flow VADs. The precise correlation between LVI and ventricular volume may differ between individual animals, because the amount of LV muscle and the irregular inflow from the LA responsible for changes in LVI can differ according to the animal’s state. Despite difficulties in analyzing original LVI, unexpected decreases in R-T impedance changes could be compared with predicted values obtained using the new algorithm, and the differences could facilitate identification of abnormal heart contraction. The actual LVI was 18 ~ 24 ohms and the actual impedance changes were 2 – 3 ohms. Abnormal LVI changes at PVC were 29.5%  ±  12.4% smaller than normal. Abnormal performance of the heart was defined as a decrease in performance to below 50%. Using this as the definition of abnormal heartbeat, the *P*-value was below 0.05; therefore the accuracy of this method appears adequate for detecting abnormal heartbeat. Distinguishing the R-T period is a crucial part of LVI measurement. To determine whether the presence or absence of PVC affects recognition of the R-T period, CO and the beginning and end of a waveform were compared. The algorithms successfully recognized the R-T period regardless of the occurrence of PVC. The hypothesis that the difference between the predicted and actual LVI values is due to a change in cardiac performance requires the assumption that the VAD maintains a constant output. Although actual VAD output can be affected by a patient’s hemodynamic state, recently developed monitoring methods could increase the accuracy of impedance prediction [17].

Although many arrhythmias were observed during the experiment, most were PVCs. PVCs showed various temporal waveforms in ECGs depending on their origin in the heart. PVCs have not been considered a critical abnormality, because they rapidly disappear, are difficult to detect, and do not show distinct symptoms in ABP and HR. The algorithm presented here shows that heart activity changes during PVCs and that these short-term changes can be detected by this algorithm. However, additional experiments are required to verify its ability to detect other types of arrhythmia.

## Conclusions

This study proposes a new approach to the analysis of heart activities by using ventricular contractions, rather than the action potentials used in conventional ECGs. Comparison of v-ECGs and impedances yielded relatively accurate values for LVI increase (R-T period) as well as effective monitoring during events such as PVCs. This may aid in overcoming the limitations of monitoring the heart and VAD by using solely flow rate and pressure, allowing more detailed monitoring of the heart for functional diagnosis. In addition, by comparing the experimental data with conventional flow, pressure, and ECG readings, the method proposed here was shown to be the most reliable means of measuring the ventricular volume, and therefore determining that of the heart. This method is expected to be applicable as a biomonitoring technique in extracorporeal circulatory systems using cannulas, such as extracorporeal membrane oxygenation (ECMO), as well as an effective method for mointoring VAD patients.

## References

[1] Slaughter MS, Singh R (2012). The role of ventricular assist devices in advanced heart failure. Rev Esp Cardiol.

[2] Lietz K, Long JW, Kfoury AG, Slaughter MS, Silver MA, Milano CA (2007). Outcomes of left ventricular assist device implantation as destination therapy in the post-REMATCH era: implications for patient selection. Circulation.

[3] Rose EA, Gelijns AC, Moskowitz AJ, Heitjan DF, Stevenson LW, Dembitsky W (2001). Long-term use of a left ventricular assist device for end-stage heart failure. N Engl J Med.

[4] Trivedi JR, Cheng A, Singh R, Williams ML, Slaughter MS (2009). Survival on the heart transplant waiting list: impact of continuous flow left ventricular assist device as bridge to transplant. Ann Thorac Surg.

[5] Frazier OH, Myers TJ, Westaby S, Gregoric ID (2003). Use of the Jarvik 2000 left ventricular assist system as a bridge to heart transplantation or as destination therapy for patients with chronic heart failure. Ann Surg.

[6] Refaat M, Chemaly E, Lebeche D, Gwathmey JK, Hajjar RJ (2008). Ventricular arrhythmias after left ventricular assist device implantation. Pacing Clin Electrophysiol.

[7] Ziv O, Dizon J, Thosani A, Naka Y, Magnano AR, Garan H (2005). Effects of left ventricular assist device therapy on ventricular arrhythmias. J Am Coll Cardiol.

[8] Bedi M, Kormos R, Winowich S, McNamara DM, Mathier MA, Murali S (2007). Ventricular arrhythmias during left ventricular assist device support. Am J Cardiol.

[9] Busch MC, Haap M, Kristen A, Haas CS (2011). Asymptomatic sustained ventricular fibrillation in a patient with left ventricular assist device. Ann Emerg Med.

[10] Refaat MM, Tanaka T, Kormos RL, McNamara D, Teuteberg J, Winowich S (2012). Survival benefit of implantable cardioverter-defibrillators in left ventricular assist device-supported heart failure patients. Card Fail.

[11] Misiri J, Jusumoto F, Goldschlager N (2012). Electromagnetic interference and implanted cardiac devices: the medical environment (part II). Clin Cardiol.

[12] Ambardekar AV, Lowery CM, Allen LA, Cannon AP, Cleveland JC, Lindenfeld J (2010). Effect of left ventricular assist device placement on preexisting implantable cardioverter-defibrillator leads. J Card Fail.

[13] Foo D, Walker BD, Kuchar DL, Thorburn CW, Tay A, Hayward CS (2009). Left ventricular mechanical assist devices and cardiac device interactions: an observational case series. Pacing Clin Electrophysiol.

[14] Kishimoto Y, Takewa Y, Arakawa M, Umeki A, Ando M, Nishimura T (2013). Development of a novel drive mode to prevent aortic insufficiency during continuous-flow LVAD support by synchronizing rotational speed with heartbeat. J Artif Organs.

[15] Choi SW, Nam KW, Lim KM, Shim EB, Won YS, Woo HM (2014). Effect of counter-pulsation control of a pulsatile left ventricular assist device on working load variations of the native heart. BioMedical Engineering OnLine.

[16] Choi SW, Park SM (2012). Analysis of left ventricular impedance in comparison with ultrasound images. Artif Organs.

[17] Kim YI, Her K, Kang SM, Choi SW (2014). Estimation of ventricular assist device outflow with the pressures in air pressure line. J Biomedical Eng Res.

[18] Pan J, Tompkins WJ (1985). A real-time QRS detection algorithm. IEEE Trans Biomed Eng.

[19] Park SM, Lee JH, Choi SW (2014). Detection of premature ventricular contractions on a ventricular electrocardiogram for patients with left ventricular assist devices. Artif Organs.

